# A Critical Review of Risk Assessment Models for *Listeria monocytogenes* in Seafood

**DOI:** 10.3390/foods13050716

**Published:** 2024-02-26

**Authors:** Ursula Gonzales-Barron, Vasco Cadavez, Juliana De Oliveira Mota, Laurent Guillier, Moez Sanaa

**Affiliations:** 1 Centro de Investigação de Montanha (CIMO), Instituto Politécnico de Bragança, Campus de Santa Apolónia, 5300-253 Bragança, Portugal; vcadavez@ipb.pt; 2 Laboratório para a Sustentabilidade e Tecnologia em Regiões de Montanha, Instituto Politécnico de Bragança, Campus de Santa Apolónia, 5300-253 Bragança, Portugal; 3 Department of Nutrition and Food Safety, World Health Organization (WHO), CH-1211 Geneva, Switzerland; 4 Risk Assessment Department, French Agency for Food, Environmental and Occupational Health & Safety (Anses), 14 Rue Pierre et Marie Curie, 94701 Maisons-Alfort, France; laurent.guillier@anses.fr

**Keywords:** systematic review, exposure assessment, simulation, fish, smoked salmon, listeriosis

## Abstract

Invasive listeriosis, due to its severe nature in susceptible populations, has been the focus of many quantitative risk assessment (QRA) models aiming to provide a valuable guide in future risk management efforts. A review of the published QRA models of *Listeria monocytogenes* in seafood was performed, with the objective of appraising the effectiveness of the control strategies at different points along the food chain. It is worth noting, however, that the outcomes of a QRA model are context-specific, and influenced by the country and target population, the assumptions that are employed, and the model architecture itself. Studies containing QRA models were retrieved through a literature search using properly connected keywords on Scopus and PubMed^®^. All 13 QRA models that were recovered were of short scope, covering, at most, the period from the end of processing to consumption; the majority (85%) focused on smoked or gravad fish. Since the modelled pathways commenced with the packaged product, none of the QRA models addressed cross-contamination events. Many models agreed that keeping the product’s temperature at 4.0–4.5 °C leads to greater reductions in the final risk of listeriosis than reducing the shelf life by one week and that the effectiveness of both measures can be surpassed by reducing the initial occurrence of *L. monocytogenes* in the product (at the end of processing). It is, therefore, necessary that future QRA models for RTE seafood contain a processing module that can provide insight into intervention strategies that can retard *L. monocytogenes*’ growth, such as the use of bacteriocins, ad hoc starter cultures and/or organic acids, and other strategies seeking to reduce cross-contamination at the facilities, such as stringent controls for sanitation procedures. Since risk estimates were shown to be moderately driven by growth kinetic parameters, namely, the exponential growth rate, the minimum temperature for growth, and the maximum population density, further work is needed to reduce uncertainties.

## 1. Introduction

*Listeria monocytogenes* is a ubiquitous microorganism that is widely distributed in the environment. Whereas soil and water are considered to be the primary sources of *L. monocytogenes* for transmission to plant material, feed, animals and the food chain, this pathogen has also demonstrated the ability to resist adverse environmental conditions and persist in the processing environment [1]. Ready-to-eat (RTE) foods that are not heat-treated or do not undergo any listericidal treatment before consumption are of significance in the transmission of foodborne listeriosis [1]. Recently, using a generic quantitative risk assessment (QRA) model, EFSA [2] compared the probability of listeriosis in the elderly population in the European Union and its link to products such as RTE fish, pâté, cooked meats, sausages, soft and semi-soft cheeses, and blanched frozen vegetables. They found out that gravad fish in normal-atmosphere packaging, and hot-/cold-smoked fish in reduced-oxygen packaging (ROP) ranked as the most high-risk products. The occurrence of *L. monocytogenes* in RTE products has been responsible for outbreaks and product recalls in the EU and the USA. For instance, as reported by the Rapid Alert System for Food and Feed (RASFF), over 40% of the seafood notifications between 2008 and 2016 were related to *L. monocytogenes* [1]. According to the latest EFSA and ECDC report [3] for the year 2022, the highest occurrences of *L. monocytogenes* were found for the categories of fish (2.6%; n = 971) and fishery products (2.5%; n = 842) sampled at the manufacturing and the distribution stages in the EU MS, with the proportion of samples exceeding the limit of the food safety criteria at distribution (100 CFU/g) generally being low (0.05% and 0.08%, respectively).

In terms of reported outbreaks, according to EU surveillance data [4], in the period between 2010 and 2020, fish and fish products (namely, crab meat, crustaceans, shellfish and molluscs, smoked fish and non-specified seafood) caused 23% of the 53 cases with strong evidence of outbreaks in the EU. Unlike the EU scenario, in the USA during the ten-year span, fish and fish products (namely, smoked fish and raw sushi) had a lower than 6% share in the 50 strong-evidence outbreaks [5]. Considering that most of the listeriosis cases occur sporadically [1,6], the results of a recent meta-analysis on case-control studies of sporadic listeriosis [7] cannot be overlooked. Combining the odds ratio (OR) outcomes from 12 primary studies, Leclercq et al. [7] found that RTE seafood presented the highest association with sporadic listeriosis, with a pooled OR of 10.95 (*p* < 0.001) for non-perinatal population, and pooled OR of 6.273 (*p* < 0.001) for the entire susceptible population (in comparison to processed meats, cheese, vegetables, fruits, and composite foods).

Various listeriosis QRA models have been produced for RTE seafood [8] in an attempt to provide guidance to reduce the occurrence of *L. monocytogenes* via practices or strategies that retard or prevent the growth of this pathogen. The objectives of this study are as follows: (i) to undertake a critical review of the published QRA models of listeriosis acquired from the consumption of RTE seafood; (ii) to contrast the control measures or strategies evaluated in the various QRA models as what-if scenarios; and (iii) to derive important lessons and recommendations for future QRA models in RTE seafood.

## 2. Materials and Methods

QRA models were retrieved through a literature search on Scopus and PubMed^®^, using 1998 as the starting year of publication. The searches of the title, keywords, and abstract were carried out on 18 May 2022, using logically connected terms ((“risk assessment” OR exposure OR quantitative microbial OR risk modelling OR modeling OR simulation* OR second-order OR “second order” OR “risk management”) AND (“*L. monocytogenes*” OR “*Listeria monocytogenes*” OR listeriosis)) properly arranged in the syntaxes of the literature search engines. Studies were considered eligible if (1) they presented a quantitative risk or exposure assessment for listeriosis linked to seafood, with explicitly indicated formulae and assumptions, and (2) they were written in English or Spanish language. The full systematic review process and extraction of information are described in the work of Gonzales-Barron et al. [8]. The present review focuses only on seafood products, which were the subject of 13 QRA models described in 14 publications [1,6,9,10,11,12,13,14,15,16,17,18,19,20].

## 3. Results

A total of 13 QRA models on seafood as a source of listeriosis were recovered in the literature search of models published between January 1998 and May 2022. Table 1 compiles the main features of the 13 QRA models, whereas Table 2 summarises the predictive microbiology models and main outcomes related to what-if scenarios and sensitivity analyses. None of the 13 QRA models comprised simulations of cross-contamination, nor did a single QRA model include a processing stage module. All the models represented short supply chains, from the end of processing or retail to table, or focused on consumption only. Three QRA models assessed the growth of *L. monocytogenes* from the end of processing until consumption [9,10,11,12], and the other three began the analysis at retail [1,6,13]. Seven out of the thirteen models solely represented the consumption module [14,15,16,17,18,19,20].

Most of the models focused on RTE seafood, except for one [19], which dealt with a traditional processed fish from Ghana. Within the RTE seafood, the majority of models (75%) estimated the risk of listeriosis from smoked fish (or salmon), while the other 25% estimated the risk associated with gravad fish. In addition, other products were investigated, such as raw seafood, preserved fish, cooked RTE crustaceans by FDA-FSIS [6], and salt-cured fishery products, as studied by Pasonen et al. [20].

More than half of the available models (9/13) represented the conditions of European countries, namely France [9,10,11], Finland [20], Ireland [18], Sweden [14], and Spain [16]—where these products are highly consumed—in addition to models that used data from the EU [1,13]. Two QRA models pertained to the risk of listeriosis in the USA’s population [6,12], and two models were not linked to any specific geographical location [15,17] (Table 1).

The QRA models that were retrieved varied in the degree of complexity in their construction. All QRA models, except two [14,19], employed predictive microbiology models, including microbial growth, survival, and competition models, and die-off and re-growth models (Table 2). The lag-phase duration of *L. monocytogenes* was considered in five models [12,13,15,17,18], whereas only three QRA models [9,10,11,13] employed time–temperature trajectories to more realistically estimate the kinetics of *L. monocytogenes* during cold storage and their shelf life (Table 1).

One model did not perform any risk estimation, as the authors targeted the estimation of the regulatory and recall risk [12], while the other twelve models considered illness as the end-point for risk estimation. The exponential dose–response function was the equation of choice for risk characterisation in nine QRA models, although they followed different approaches, namely the dose–response models of Pouillot et al. [21] (used by Pérez-Rodriguez et al. [13], and modified versions by Fritsch et al. [11] and EFSA-BIOHAZ [1]), FAO-WHO [15] (used by themselves [15] and Garrido et al. [16]), FDA-FSIS [6] (used by themselves), Pasonen et al. [20] (used by themselves), and Buchanan et al. [22] (used by Lindqvist and Westöö [14]). The early Weibull–gamma model proposed by Farber et al. [24] was used in the QRA models of Dass [18] and Bomfeh [19], whereas the beta-Poisson model of Haas et al. [23] was employed in the QRA model of Gospavic et al. [17] (Table 1).

Except for Bomfeh [19], all seafood QRA models assessed the effect of what-if scenarios on the final risk. A sensitivity analysis of response variables, such as *L. monocytogenes’* concentration at consumption, regulatory and recall risk, or final risk measures, was undertaken in 38.0% of the models [1,9,10,12,14,18] (Table 2).

## 4. Discussion

Listeriosis QRA models in seafood focused mainly on RTE smoked and gravad fish (i.e., generic fish, salmon, or trout), because these are products that have considerable public health implications regarding listeriosis, for the following reasons: (1) they are not heat-treated; (2) they are given a relatively long shelf-life; (3) they are mostly vacuum-packed—which does not preclude *L. monocytogenes* growth; and (4) they are generally eaten with no prior cooking. Many reports and surveys have indicated that seafood products are frequently contaminated with *L. monocytogenes* [25]. A recent genomic-based epidemiological study [26] determined that, from 2018 to 2020, 27% of all listeriosis cases in Germany with suspected food vehicles were caused by smoked or gravad salmon products. These authors demonstrated that, despite the considerable efforts that have been made to improve the safety of smoked fish, outbreaks linked to the presence of *L. monocytogenes* at infective levels in these seafood products continue to occur. Controlling *L. monocytogenes* in smoked fish is challenging because this pathogen is widely distributed in a variety of environments, including natural ones [27,28], and processing facilities [29]. This underscores the need to assess new technological interventions, post-lethality treatments, and intensified sanitation programmes to reduce the risk of listeriosis in RTE seafood. 

### 4.1. Risk Factors at Processing

Apart from *L. monocytogenes* contamination in raw materials (fish) as a primary source, smoked fish can acquire the pathogen from contact with surfaces in the processing environment. Moreover, the fate of *L. monocytogenes* is variable along the processing steps of evisceration and filleting [30,31], brining [32], smoking [33], and slicing [34]. Nevertheless, despite the extensive data that are available, none of these effects has been simulated in the seafood QRA models that were retrieved. The first QRA model of the broadest scope was that of Pouillot et al. [9,10], which followed the cold-smoked salmon supply chain from the end of processing until consumption. Such a model was innovative in the following ways: (1) it used a Jameson effect to account for the inhibitory effect of the background microflora on *L. monocytogenes* in the product within the vacuum-packaged atmosphere; (2) it used dynamic time–temperature profiles to represent realistic temperature oscillations between cold storage at the end of processing and home refrigeration. The other two QRA models represented the supply chain from the end of processing: Fritsch et al.’s [11] model, which has the same structure as Pouillot et al.’s [9,10] model, but was refined by the introduction of phenotypic growth characteristics of *L. monocytogenes* according to subgroup and virulence properties; and Chen et al.’s [12] model, which did not proceed to the risk characterisation stage.

In relation to the assessment of post-lethality treatments, such as product reformulation involving the application of lactate or diacetate, nisin, or specific starter cultures, these were not assessed in any of the QRA models that were retrieved, except in Chen et al.’s model [12], where they pointed out that the addition of nisin (5–20 ppm) is far more effective than the addition of 2% potassium lactate plus 0.14% sodium diacetate in decreasing the overall risk of a sampled lot that was found positive for *L. monocytogenes* (67–95% reduction versus 6% reduction, respectively). Although the QRA models of Pouillot et al. [9,10], Fritsch et al. [11], Pérez-Rodríguez et al. [13], and Gospavic et al. [17] were equipped with Jameson-effect models that were able to characterise the inhibitory effect of lactic acid bacteria on *L. monocytogenes*, none of these QRA models evaluated scenarios related to the addition of ad hoc cultures of lactic acid bacteria (Table 2). Although the cold-smoked fish model of FAO-WHO [15] did not employ any Jameson-effect competition model, it considered the effect of indigenous lactic acid bacteria, assuming that, at high concentrations, they can suppress the growth of *L. monocytogenes*. This model demonstrated the ability of cold-smoked fish to support the growth of *L. monocytogenes*. Even under the optimistic assumption that the growth rate inhibition of *L. monocytogenes* due to the growth of lactic acid bacteria is 95%, the listeriosis cases per thousand people would be ~70-fold greater than if cold-smoked fish did not support their growth (Table 2).

### 4.2. Cross-Contamination in Processing Plants

Abundant literature has demonstrated that cross-contamination can occur during the different processing stages of RTE seafood. During head-cutting, evisceration, and filleting, there are many opportunities for *L. monocytogenes* to be transferred from the exterior of fish to the cut surfaces of fillets or sides [30,35]. At this stage of processing, the flesh areas of fish can be inoculated by contact with the contaminated skin sides of fillets, filleting tables, and knives and gloves. For instance, Dass [18] detected *L. monocytogenes* types c and b on filleting boards, deboning pins, conveyor belts, and knives. In another study, Chen et al. [31] monitored the contamination of *L. monocytogenes* in catfish fillets and in environmental samples collected from various areas of the processing plant. They isolated serotype 1/2b (47%) from trimming boards, conveyor belts, and holding tables, and found that conveyors were contaminated with *L. monocytogenes* in a total of 16.6% of the samplings (6/36). In a processing facility of gravad salmon in Brazil, Cruz et al. [36] 80% of swabs of *L. monocytogenes* from handlers were found to be positive, and 25% from knives and tables. Lundén et al. [37] explained that the contamination on tables and cutting surfaces can adhere strongly within a short period of time. This suggests that filleted fish become contaminated during the first stages of processing. 

Slicing machines can be regarded as a source of *L. monocytogenes’* contamination [34]. Di Ciccio et al. [38] repeatedly isolated *L. monocytogenes* serotypes 1/2a and 1/2b from slicer belts, distribution trays, slicing machines, and slicing covers for three years in a smoked-salmon production facility. Out of the 95 tested environmental samples, slicing machines (37%) and working tables (43%) had the highest frequencies of detection. In the USA, in a processing plant of catfish fillets, Chen et al. [31] determined that 15% (7/45) of the samples, monitored at skinning, slicing, and blending equipment, were contaminated with *L. monocytogenes*. 

The review published by Jami et al. [25] showed that drains and floors, as non-direct food-contact surfaces, are the most frequently contaminated sources, with prevalences of 2–80% and 1–53%, respectively. Drains and floors may, therefore, represent niches of contamination. Many authors have also found that cross-contamination can occur during packaging from surfaces that are in direct contact with the food being packaged. In cold-smoked salmon plants, Autio et al. [39], Vogel et al. [40], Klaeboe et al. [41], Nakamura et al. [42], Thimothe et al. [43], and Hu et al. [44] recovered *L. monocytogenes* from direct food-contact surfaces of packaging equipment at frequencies of 20/84, 140/818, 23/155, 9/101, 6/125, and 5/344, respectively.

Nonetheless, despite the actual environmental contamination that occurs in processing plants, none of the 13 QRA models assembled cross-contamination modules that could help assess the effects of implementing more stringent controls for environmental monitoring programmes, good manufacturing practices, and standard operation procedures for sanitation.

### 4.3. Shelf-Life and Risk Factors at Retail and Home

As the seafood QRA models had a short scope, the typical scenarios that were assessed were those related to reductions in *L. monocytogenes*’ initial prevalence/concentration, storage temperature, time of consumer storage, and shelf-life (Table 2). Vacuum-packaging is widely used in the smoked/gravad fish industry as it delays the proliferation of aerobic spoilage bacteria and minimises oxidative reactions. However, although vacuum-packaging is used to extend the shelf-life of these products, microaerophilic or facultative anaerobic microorganisms, such as *L. monocytogenes*, may thrive under such condition, and an extended shelf-life may provide sufficient time for the pathogen to increase to infective levels. The EFSA BIOHAZ generic QRA model [1] clearly made this point by showing that, across three RTE fish products (cold-smoked, hot-smoked and gravad fish), there is no strong difference in the probability of a product exceeding 100 CFU/g at the time of consumption between normal packaging (0.066–0.112) and reduced-oxygen packaging (0.040–0.115; Table 2). Within this context, many of the QRA models tested what-if scenarios with a shorter time of storage before consumption. Researchers obtained different estimates of the degree of reduction in the number of listeriosis cases for smoked fish.

Decreasing the consumption time of unopened packages to a maximum of 7 days at cold storage led to reductions in the cases of listeriosis of 80% for cold-smoked fish (FAO-WHO [15]; baseline 14 days), 80% for packaged cold-/hot-smoked fish and gravad fish (Pérez-Rodríguez et al. [13]; baseline not clear), 63% for cold-smoked salmon (Pouillot et al. [10]; baseline 32 days maximum), 45% for smoked trout (Garrido et al. [16]; baseline 30 days), and 15% for smoked salmon (Garrido et al. [16]; baseline 30 days). Reducing the shelf life to 14–15 days for cold-smoked salmon was estimated to reduce the listeriosis cases by 77% (Pouillot et al. [9,10]; baseline 32 days) and the risk of illness by 64% (Gospavic et al. [17]; baseline 28 days). FDA-FSIS [6] estimated that by reducing the maximum home storage time from 45 to 30 days, the mean cases of listeriosis would be reduced by 38% in the elderly population (Table 2). 

Shelf-life reduction therefore appears to be a good strategy to decrease the risk of listeriosis, although its effectiveness can be surpassed by effectively maintaining the product at 4.0–4.5 °C during home storage, as attested in the what-if scenarios presented by Dass [18], Garrido et al. [16], Pérez-Rodríguez et al. [13], and Pouillot et al. [9,10]. Furthermore, in a sensitivity analysis carried out on the annual risk of illness in the high-risk population linked to smoked salmon, the temperature in the consumer’s fridge was more determinant of risk (r = 0.13) than the time spent in the consumer’s fridge (r = 0.06) [18]. A comparable rank correlation coefficient for storage temperature (r = 0.177) was estimated by Chen et al. [12]. In a Bayesian QRA model [20], although no comparison was made between a temperature-lowering scenario with a shelf-life reduction scenario, the authors underscored the importance of maintaining a cold temperature at the domestic level by predicting that if the home mean storage temperature decreased from 7 °C to 3 °C, the median cases of listeriosis per 100,000 people would decrease by 70% for the elderly population and 40% for the working-age population (Table 2).

Pasonen et al.’s [20] model was different from the other models in that it allowed for the possibility of continuing consumption of the same (contaminated) package of cold- smoked and salt-cured salmon, rather than assuming independent consumption days, and in that the model was not solved as a forward problem according to Monte Carlo simulation. This QRA model was built on a Bayesian, two-state, Markov chain approach consisting of three modules: a module for occurrence data, a module for consumption data, and a predictive model for the total number of listeriosis in the population. As a Markov-chain Monte Carlo (MCMC) simulation, bottom-up and top-down approaches are combined, and thus all unknown parameters can be jointly estimated from a single compact model. As a result, Pasonen et al. [20] could estimate the uncertainty distribution of the parameters, truly reflecting the information contained in the data. The r parameter of the exponential dose–response model was estimated from Finnish data by using the reported number of listeriosis cases to calibrate the dose–response function for the target populations. Nonetheless, in this calculation, it was assumed that all these listeriosis cases resulted from the consumption of the smoked fish, because the attribution of other sources was not modelled. This fact does not invalidate the MCMC approach proposed by Pasonen et al. [20], since the parameter(s) of the dose–response function could be assumed to be known. Although it demands a more complex programming, Bayesian inference features many advantages such as utilising the whole data set jointly, handling censored values, taking uncertainty into account, and the possibility of using prior knowledge.

Decreasing the initial mean prevalence or concentration of *L. monocytogenes* was another of the frequent what-if scenarios, which, in most of the QRA models of smoked salmon, turned out to be at least as effective in reducing the risk of listeriosis as maintaining the consumer’s fridge temperature at 4 °C. This was observed in the models of Pouillot et al. [9,10], Chen et al. [12], Pérez-Rodríguez et al. [13], Gospavic et al. [17], Dass [18], and Lindqvist and Westöö [14], and will be described as follows. In the hypothetical scenario where the prevalence of contaminated packages of cold-smoked salmon decreased from 1.0 to 0.25, the number of listeriosis cases would drop by 75% (Pouillot et al. [9,10]). Chen et al. [12] estimated that reducing the initial prevalence to half would cause a reduction of 45% in the regulatory and recall risk. Pérez-Rodríguez et al. [13] predicted that decreasing the maximum initial concentration of *L. monocytogenes* by 2.0 log would ensure a drop in listeriosis cases by >99.9%; Gospavic et al. [17] estimated that reducing the mean initial concentration from 25 CFU/g to 4 CFU/g would reduce the risk of listeriosis by 67%. In line with the QRA models presented above, looking at sensitivity analysis outcomes, Dass [18] also showed that the concentration of *L. monocytogenes* at retail was a stronger determinant (r = 0.97) of the annual risk of illness in the high-risk population than the temperature/time in the consumer’s fridge (r = 0.06–0.13). Similarly, Chen et al. [12] estimated that the initial contamination level (r = 0.404) was a stronger determinant of the regulatory and recall risk than the storage temperature (r = 0.177). Without providing coefficients of correlation, Lindqvist and Westöö [14] indicated that the variables impacting the annual risk of listeriosis, in decreasing order, were as follows: the initial counts of *L. monocytogenes* at retail; the prevalence of *L. monocytogenes*; the serving size and proportion of virulent strains (Table 2).

The results of these short-scope QRA models have shown, as a whole, that even when storage temperatures can be kept as low as 3–4 °C by the consumers, *L. monocytogenes* can proliferate in smoked/graved fish. However, reducing the initial contamination or ensuring that prevalence is low at the beginning of retail (or end of processing) would result in a significantly lower risk of listeriosis. Therefore, a robust processing module should be built to represent the strategies, combination of strategies, and/or sanitation control schemes leading to reductions in the prevalence/concentration of *L. monocytogenes*.

### 4.4. Microbial Growth Kinetic Parameters as Drivers of the Final Risk

Interesting outcomes from Pouillot’s QRA model included the high impact of the kinetic parameters of both *L. monocytogenes* and background microflora on both the concentration of *L. monocytogenes* in contaminated servings [9] and the listeriosis cases in the reference population [10]. Right after the initial *L. monocytogenes* prevalence (*p* = 10^−20^) and the mean temperature at retail phase (*p* = 10^−20^), the concentration of *L. monocytogenes* in contaminated servings was highly sensitive to the minimum temperatures regarding the growth of *L. monocytogenes* (*p* = 10^−8^) and the background microflora (*p* = 10^−6^), followed by the reference growth rates at 25 °C of *L. monocytogenes* (*p* = 0.015) and the background microflora (*p* = 0.025), and the maximum population density (*p* = 0.008). Likewise, the listeriosis cases in the reference population were heavily impacted by the mean and the standard deviation of the maximum population density (*p* = 10^−137^; *p* = 10^−76^), the reference growth rates at 25 °C of *L. monocytogenes* (*p* = 10^−101^) and the background microflora (*p* = 10^−8^), and the minimum temperature for the growth of *L. monocytogenes* (*p* = 10^−12^). Likewise, Chen et al. [12] found that, after the initial contamination level (r = 0.404), important determinants of the recall risk included the microbial kinetic parameters of exponential growth rate at 25 °C (r = 0.275) and the minimum temperature for growth (r = −0.169). 

The distribution of the minimum temperature for growth was also demonstrated to have an impact on the mean concentration of *L. monocytogenes* in servings in Fritsch et al.’s [11] model. The ability to multiply in the cold was correlated with the presence of a genetic marker for cold adaptation. The authors looked for this marker in a collection of strains representative of smoked salmon at the distribution stage [44]. By defining two distributions of minimum temperature for growth for slow-growing strains and fast-growing strains, Fritsch et al. [11] showed that the mean exposure of the consumer was two times more important in the high-growth groups (25 CFU/g) compared to the low-growth groups (13 CFU/g). However, the importance of the type of strain on exposure was less significant for the risk of listeriosis than the presence of virulence markers.

In addition to the QRA model of Pouillot et al. [9,10], two other models ascertained that the maximum population density of *L. monocytogenes* is not a parameter of minor importance. Whereas Chen et al. [12] found a certain association with the risk recall (r = 0.053), EFSA BIOHAZ [1] determined that the listeriosis risk was very sensitive to the maximum population density, and quantified that a shift in 0.5 log CFU/g can double the estimated risk (Table 2). In order to obtain more precise estimates of the risk, it is therefore important to reduce the uncertainties regarding the characteristics of *L. monocytogenes*, including the parameters associated with the exponential growth rate, the nominal minimum temperature for growth, and the maximum population density.

### 4.5. Models’ Availabiltity 

The most widespread use of risk assessment approaches involves the sharing and description of models [45]. As we have recently shown for other foods [8], most QMRA models for seafood are often unavailable. Details of the software used and links describing the available models are provided in Table S1 of the Supplementary Material of this article.

## 5. Conclusions

Eighty-five percent of the QRA models focused on cold-smoked/gravad fish because of the considerable and continuous public health implications of the seafood products. Despite the availability of *L. monocytogenes* data on stages such as filleting, brining, smoking, and slicing, none of the QRA models contained a processing module; therefore, cross-contamination events were not represented. Since secondary contamination can occur in the processing plants from equipment and environmental elements, the most relevant opportunities for cross-contamination during processing should be identified and modelled. Thus, in addition to cold chain (distribution and retail) and consumption stages, which have been represented by most QRA models, a future smoked/gravad fish model should also comprise a processing module that is robust enough to allow for an assessment of the following: (1) intervention strategies that can retard the growth of *L. monocytogenes*, such as the application of bacteriocins (nisin), suitable starter cultures, and/or organic acids; (2) control measures that can reduce the frequency of cross-contamination events, such as the implementation of more stringent controls for raw materials, environmental monitoring programs, and/or sanitation procedures. Given the strong evidence of the inhibitory effect of background microflora on *L. monocytogenes* in vacuum-packed RTE smoked fish, predictive microbiology models that describe microbial competition should be employed after the reduced-oxygen packaging stage. Furthermore, since the growth kinetic parameters of *L. monocytogenes* and microflora have been demonstrated to have a heavy influence on the estimated risk of listeriosis, efforts should be made to accurately model such growth kinetic parameters.

## Figures and Tables

**Table 1:** Features of quantitative risk assessment models of *L. monocytogenes* regarding the consumption of seafood products by scope.

Scope	Food	RTE	Cross-Conta-mination	DR—End-Point	Type of DR Model	DR Sub-Populations	Strain Variability	Temp Profiles/ Lagtime	Country	Source
End-processing-to-table	Cold-smoked salmon	Yes	No	Exp—I	Pouillot et al. [10]	Multiple	NA	Yes/No	France	Pouillot et al. [9,10]
	Cold-smoked salmon	Yes	No	Exp—I	Fritsch et al. [11]: r values from Pouillot et al. [21] were re-scaled to three diff-erent groups of virulence (according to CCs)	General	Specific prevalence for each LM genotypic sub-group (CC) in Europe; two different distributions for Tmin to represent “low-growing” and “high-growing” strains; three virulence levels in the DR r values	Yes/No	France	Fritsch et al. [11] (model based on Pouillot et al. [9,10] integra-ting genomic data)
	Cold-smoked salmon	Yes	No	None	NA	NA	Variable proportion of contaminated packages and growth kinetics parameters according to LM serotypes 1/2a, 1/2b, and 4b	No/Yes	USA	Chen et al. [12]
Retail-to-table	Various: smoked seafood, raw seafood, preserved fish, cooked RTE crustaceans	Yes	No	Mouse Epi—I	FDA-FSIS [6]	Multiple	Variability in the virulence of different strains represented in DR	No/No	USA	FDA-FSIS [6]
	Packaged cold-/hot-smoked fish and gravad fish	Yes	No	Exp—I	Pouillot et al. [21]	Multiple	Challenge test data from a mixture of strains; h0 distribution of variability in physiological state of cells; variability in strain virulence and in susceptibility across population subgroups	Yes/Yes	EU	Pérez-Rodríguez et al. [13]
	Cold-, hot-smoked fish, gravad fish	Yes	No	Exp—I	EFSA BIOHAZ [1] based on Pouillot et al. [21]	Multiple (sex/age group)	Challenge test data from a mixture of strains; strain virulence and host susceptibility explicit in r distribution	No/No	EU	EFSA BIOHAZ [1]
Consumption	Smoked/gravad salmon/rainbow trout	Yes	No	Exp—I	Buchanan et al. [22]	General	All strains are virulent vs. a proportion of virulent strains	No/No	Sweden	Lindqvist and Westöö [14]
	Cold-smoked fish	Yes	No	Exp—I	FAO-WHO [15]	High-risk/low-risk	Strain diversity implicit in r	No/Yes	Non-specific	FAO-WHO [15]
	Smoked fish and sliced cooked ham	Yes	No	Exp—I	FAO-WHO [15]	High-risk/low-risk	Strain diversity implicit in r	No/No	Spain	Garrido et al. [16]
	Cold-smoked salmon	Yes	No	BP—I	Haas et al. [23]	General	NA	No/Yes	Non-specific	Gospavic et al. [17]
	VP cold-smoked salmon	Yes	No	WG—I	Farber et al. [24]	High-risk/low-risk	Challenge test data from a mixture of strains	No/Yes	Ireland	Dass [18]
	Traditional processed fish	No	No	WG—I	Farber et al. [24]	High-risk/low-risk	NA	No/No	Ghana	Bomfeh [19]
	Cold-smoked and salt-cured fishery products	Yes	No	Exp—I	Pasonen et al. [20]	High-risk/low-risk	NA	No/No	Finland	Pasonen et al. [20]

DR: dose–response; Exp: exponential model; Mouse-Epi: mouse epidemiological model; I: illness endpoint; D: death endpoint; NA: not addressed in the study.

**Table 2:** Predictive microbiology models and main outcomes related to what-if scenarios and sensitivity analysis from quantitative risk assessment models of *L. monocytogenes* (LM) from consumption of seafood products.

Scope	Food	Predictive Microbiology Models	What-If Scenarios	Sensitivity Analysis	Model Complexity	Source
End processing-to-table	Cold-smoked salmon	Growth (Jameson effect LM and background microflora, growth square root models for LM and background microflora)	**EXPOSURE ASSESSMENT**: (1) Reducing theoretical shelf-life from 28 days to 15 days reduced mean LM/g in contaminated servings by 10%; (2) the baseline scenario of 21.4% of shelf lives at home being longer than 7 days was compared to a scenario of consumption within 7 days maximum, which reduced the mean LM/g by 10%; (3) better refrigeration at retail, reducing the mean temperature from 5.6 to 4 °C, reduces the mean LM counts by 19%; (4) better refrigeration at home, reducing the mean temperature from 7 to 4 °C, reduces the mean LM counts by 36%; (5) a lower initial concentration, from 0.46% of values above 1 CFU/g to a distribution truncated at 1 CFU/g, reduces the mean LM counts by 8%. **RISK ASSESSMENT**: Output—Listeriosis cases compared to a base 100 for the baseline model: (1) shelf-life 15 days = 23; (2) prevalence of LM to a quarter= 25; (3) mean home refrigerator temperature 4 °C = 34; (4) consumed 7 days after purchase = 37; (5) prevalence of LM to a half = 50; (6) mean retail temperature at 4 °C = 67.	**EXPOSURE ASSESSMENT: Output—concentration of LM in contaminated servings**: (1) total duration at the consumer phase (*p* = 10^−30^); (2) mean temperature at the consumer phase (*p* = 10^−20^); (3) initial LM counts (*p* = 10^−20^); (4) mean temperature at retail phase (*p* = 10^−14^); (5) total duration of retail phase (*p* = 10^−8^); (6) Tmin for growth (*p* = 10^−8^); (7) Tmin microflora (*p* = 10^−6^); (8) initial background flora counts (*p* = 0.002); (9) serving size (*p* = 0.003); (10) MPD (*p* = 0.008); (11) ref. GR at 25 °C (*p* = 0.015); (12) ref. GR of flora at 25 °C (*p* = 0.025). **RISK ASSESSMENT: Output—listeriosis cases in the reference population**: (1) r value of DR model (*p* = 10^−300^); (2) SD (MPD) (*p* = 10^−137^); (3) ref. of GR 25 °C for LM (*p* = 10^−101^); (4) MPD of LM (*p* = 10^−76^); (5) Tmin of LM (*p* = 10^−12^); (6) GR of flora 25 °C (*p* = 10^−8^); (7) prevalence of LM (*p* = 10^−6^); (8) servings/year (*p* = 10^−2^).	medium: complex predictive microbiology model, a new method for solving growth under dynamic temperature profiles, was proposed.	Pouillot et al. [9,10]
	Cold smoked salmon	Growth (Jameson effect LM and background microflora, growth square root models for LM and background microflora)	Baseline predicted 978 listeriosis cases after consumption of 50 g cold-smoked salmon with an initial LM prevalence of 10.4%, considering a single prevalence distribution. (1) Taking into account specific prevalences for each LM genotypic sub-group lowered the listeriosis cases to 574. (2) A total of 97% of listeriosis cases were caused by the hypervirulent group, despite their low prevalence (12.6%) in contaminated salmon. Inversely, the most prevalent (hypovirulent) group (51.7%) was responsible for only 0.02% of the listeriosis cases. (3) The effect of the low/high-growth strains (two distributions for Tmin) was lower than the effect of the virulence: mean exposure from the high-growth LM group was 25 CFU/g compared to the low-growth groups (13 CFU/g).	NA	Medium: Same as Pouillot et al. [9,10] but with the further complexity of adding phenotypic characteristics of LM by subgroup and the virulence properties of LM.	Fritsch et al. [11] (model based on Pouillot et al. [9,10], integrating genomic data)
	Cold-smoked salmon	Growth models (Buchanan, Gompertz and Baranyi as primary models, and secondary square root model); and Die-off and re-growth models (Weibull-Buchanan, Weibull-Gompertz and Weibull-Baranyi)	End point of the model is the *regulatory and recall risk* (RRR), defined as the overall risk of a lot sampled found positive for LM. (1) Treatment of salmon with 5 or 20 ppm nisin reduced RRR to 0.109 or 0.017 (in comparison to baseline RRR of 0.333); (2) reducing prevalence to half decreased RRR to 0.182; (3) the use of inhibitors (2% potassium lactate + 0.14% sodium diacetate) slightly reduced RRR to 0.313; (4) keeping cold storage below 5 °C did not reduce RRR.	**Output—regulatory and recall risk**: (1) initial contamination level (r = 0.404); (2) GR at 25 °C (r = 0.275); (3) storage temperature (r = 0.177); (4) Tmin (r = −0.169); (5) MPD (r = 0.053)	Medium: Uncertainty and variability are separated; the die-off and/or growth kinetics are too compartment-alised.	Chen et al. [12]
Retail-to-table	Various: smoked seafood, raw seafood, preserved fish, cooked RTE crustaceans	Growth (linear model, EGR5 square root models)	(1) For cold-smoked salmon, reducing the max. home storage time from 45 to 30 days reduces the mean cases by 38% in the elderly population.	NA	Medium: Various foods	FDA-FSIS [6]
	Packaged cold-/hot-smoked fish and graved fish	Growth (Baranyi model with Jameson effect LM and LAB, EGR5 square root model and effect of lactate)	(1) Decreasing the maximum initial LM concentration by 2 log decreases listeriosis cases per million servings in >99%; (2) decreasing time to consumption by 25% decreases listeriosis by 80%; (3) decreasing 1–2 °C in the dynamic temperature profiles reduces cases by 75%; (4) including lag time in the model has no effect on listeriosis cases.	NA	Medium: Dynamic time–temperature profiles from retail to consumption, and microbial competition models used were solved with the RK4 algorithm.	Pérez-Rodríguez et al. [13]
	Cold-, hot-smoked fish, gravad fish	Growth (Rosso model, EGR 5 °C)	(1) Across the 3 RTE fish products, there is no strong difference in the probability of a product exceeding 100 CFU/g at the time of consumption between normal packaging (0.066–0.112) and reduced-oxygen packaging (0.040–0.115); (2) in both reduced-oxygen and normal packaging, hot-smoked fish presented with a higher probability of exceeding 100 CFU/g at the point of consumption (0.115, 0.112) than cold-smoked fish (0.080, 0.074) and gravad fish (0.047, 0.066).	Risk is very sensitive to MPD. A shift in 0.5 log CFU/g can double the estimated risk. However, a sensitivity analysis was conducted, taking various RTE food classes into account.	Low: Generic model; only demands some knowledge of R software to utilise it	EFSA BIOHAZ [1]
Consump-tion	Smoked/gravad salmon/rainbow trout	NA	(1) The minimum level of LM resulting in a risk of illness greater than 10^−7^ or 10^−8^ was 25 or 2 CFU/g; (2) if the assumption that all strains are virulent is reduced to 1–10%, the annual listeriosis cases are reduced by 84% in the both high-risk and the low-risk populations.	**Output—annual risk of illness**: ranked as initial LM counts, prevalence, serving size, and proportion of virulent strains	Low	Lindqvist and Westöö [14]
	Cold-smoked fish	Growth (LM growth model affected by LAB growth, square root model for GR as a function of temperature, pH, aw, un-dissociated lactic acid)	(1) If the LM growth rate inhibition due to LAB growth is between 80 and 100%, the increase in listeriosis per 100,000 people is 684-fold in the overall population, in comparison to the baseline scenario of no growth in LM between purchase and consumption; (2) if the LM growth rate inhibition due to LAB growth is 95%, the increase in listeriosis per 100,000 people is 67-fold in the overall population in comparison to the no growth in the LM baseline scenario; (3) reducing the mean shelf-life of smoked fish from 14 to 7 days results in an 80% reduction in listeriosis.	NA	Medium: Relative lag time concept for LM and LAB	FAO-WHO [15]
	Smoked fish (salmon and trout)	Growth (logistic model without delay, growth cardinal model)	(1) Reducing home storage time from a max of 30–7 days reduces the annual cases by 15% for salmon and 45% for trout; (2) if all domestic temperatures had a mean temperature of 4.5 °C, the mean annual cases are reduced by 65% for salmon and by 70% for trout; (3) Combining the two measures above reduces the mean annual cases by 75% for salmon and 87% for trout; (4) If, at purchase, LM counts do not exceed 100 CFU/g (truncating the baseline N~(1.01, 0.71) for smoked salmon and N~(1.35, 1.40) for smoked trout, the mean annual cases would decrease by 22% in salmon and 99% in trout.	NA	Low	Garrido et al. [16]
	Cold-smoked salmon	Growth (Baranyi model with Jameson effect LM and background microflora, extended GR models for LM and LAB as a function of temperature, pH, aw, un-dissociated lactic acid, undissociated diacetate, phenols, dissolved CO_2_ and nitrite)	(1) At a mean initial LM count of 4 CFU/g, reducing the time of consumption from 28 to 14 days reduces the risk of illness by 64%; (2) if the mean time of consumption is 14 days, reducing the mean initial counts from 25 CFU/g to 4 CFU/g reduces the risk of illness by 67%.	NA	Medium: stochastic fluctuations in the GR of LM are taken into account by using white noise and the Winner process.	Gospavic et al. [17]
	Vacuum-packed cold-smoked salmon	Growth (Baranyi model, growth square root model)	(1) If initial LM counts at retail (1–1000) are truncated at >100 CFU/g, the risk of illness would reduce by 0.3/0.9 log (high-risk and low-risk populations); (2) reducing the maximum consumer shopping time from 3 h to 30 min reduces risk of illness by 0.8/0.8 log; (3) reducing consumer storage days from 21–30 to 7–15 days reduces risk of illness by 0.5/0.6 log; (4) fixing storage temperature from 3–10 °C to 4 °C reduces risk of illness by 1.0/1.1 log; (5) if LM counts are not higher than 2 log CFU/g and the maximum shopping time is reduced to 30 min, reducing consumer storage days to 7–15 days and storage temperature to 4 °C reduces risk of illness by 1.32/1.39 log.	**Output—annual risk of illness in the high-risk population**: (1) LM counts at retail (r = 0.97); (2) temperature in consumer fridge (r = 0.13); (3) time in consumer fridge (r = 0.06).	Low: Lag: Baranyi model with bacterial adaptation	Dass [18]
	Traditional processed fish	NA	NA	NA	Low	Bomfeh [19]
	Cold-smoked and salt-cured fishery products	Growth (logistic growth model, growth cardinal parameter model as a function of temperature, salt content, pH and phenolic compounds)	(1) If home storage temperature decreased from 7 °C to 3 °C, the median cases of listeriosis per 100,000 elderly population would decrease by 70%; (2) if home storage temperature decreased from 7 °C to 3 °C, the median cases of listeriosis per 100,000 working-age population would decrease by 40%.	NA	High: Parameters, including r, were estimated from a Bayesian model consisting of three linked modules: a model for the occurrence data, a model for the consumption data and a predictive model for the total number of cases in the population. The current model takes into account the possibility of continuing consumption of the same (contamina-ted) package of CSS/SCS, rather than assuming independent consumption days.	Pasonen et al. [20]

aw: water activity; LPD: lag-phase duration; RLT: relative lag time; MPD: maximum population density; GR: maximum growth rate; EGR_x_: exponential growth rate at x °C; LAB: lactic acid bacteria; LAC: lactic acid concentration; RR: risk reduction; r: Pearson’s correlation coefficient; NA: not addressed in the study.

## Data Availability

Data are contained within the article. The BibTeX file containing records used in this systematic review is available.

## Supplementary Materials

The following supporting information can be downloaded at: https://www.mdpi.com/article/10.3390/foods13050716/s1: Table S1: Accessibility to published listeriosis QRA models for seafood products.
